# Viscoelastic biomarker for differentiation of benign and malignant breast lesion in ultra- low frequency range

**DOI:** 10.1038/s41598-019-41885-9

**Published:** 2019-04-05

**Authors:** Alireza Nabavizadeh, Mahdi Bayat, Viksit Kumar, Adriana Gregory, Jeremy Webb, Azra Alizad, Mostafa Fatemi

**Affiliations:** 1 Department of Physiology and Biomedical Engineering, Mayo Clinic College of Medicine, Rochester, Minnesota USA; 2 Biomedical Informatics and Computational Biology, University of Minnesota Rochester, Rochester, Minnesota, USA; 3 Department of Radiology, Mayo Clinic College of Medicine, Rochester, Minnesota USA

## Abstract

Benign and malignant tumors differ in the viscoelastic properties of their cellular microenvironments and in their spatiotemporal response to very low frequency stimuli. These differences can introduce a unique viscoelastic biomarker in differentiation of benign and malignant tumors. This biomarker may reduce the number of unnecessary biopsies in breast patients. Although different methods have been developed so far for this purpose, none of them have focused on *in vivo* and *in situ* assessment of local viscoelastic properties in the ultra-low (sub-Hertz) frequency range. Here we introduce a new, noninvasive model-free method called Loss Angle Mapping (LAM). We assessed the performance results on 156 breast patients. The method was further improved by detection of out-of-plane motion using motion compensation cross correlation method (MCCC). 45 patients met this MCCC criterion and were considered for data analysis. Among this population, we found 77.8% sensitivity and 96.3% specificity (p < 0.0001) in discriminating between benign and malignant tumors using logistic regression method regarding the pre known information about the BIRADS number and size. The accuracy and area under the ROC curve, AUC, was 88.9% and 0.94, respectively. This method opens new avenues to investigate the mechanobiology behavior of different tissues in a frequency range that has not yet been explored in any *in vivo* patient studies.

## Introduction

Almost one century ago D’Arcy Thomson proposed that the spatiotemporal alterations in tissue mechanics inevitably alter its mechanoenvironemtal properties. These local biphasic mechanical properties, such as stiffness and fluidity, determine the system response to generated forces. This theory has been accepted widely^1–7^.

Rheological techniques are rarely used for medical diagnosis of living tissue due to the invasive nature of rheological tests such as indentation experiments, torsional resonators and oscillatory shear testing devices^8–10^. These methods do allow for exploration of the viscoelastic properties of the medium in a wide range of frequencies, including the ultra-low frequency range (less than 1 Hz). Although a rheometer can assess the viscoelastic behavior of a medium in ultra-low frequency ranges, it is a time consuming and cumbersome process to use it in such a low frequency range^11–13^.

Over a decade ago, non-invasive elastography methods were developed with the capability of remotely inducing shear waves in tissue and measuring its elasticity by recording the deflection response by MRI^14^ and ultrasound^15^. Later the *in vivo* application of elastography in measuring the stiffness of different tissues and organs like liver^16–18^, breast^19,20^, brain^21,22^, heart^23,24^ and muscle^25,26^ were reported. Although elasticity measurements were the focus of these efforts, a few studies considered the estimation of both elasticity and viscosity of *in vivo* tissues^20,22,27,28^.

While it is possible to use shear wave elastography methods for *in vivo* cases, the frequency range to explore the viscoelasticity of tissue is much narrower than with rheology methods^20,22,27^. With shear wave elastography methods, reaching the ultra-low frequency range is almost impossible because there is always a tradeoff between the resolution of the resulting map and the frequency of vibration and shear waves^29^.

In this paper, we introduce the noninvasive, Loss Angle Mapping (LAM) method, which is based on measuring the local displacement and strain behaviors under constant stress as a function of frequency. Technical details were explained in our previous work^30^. The LAM method can monitor the viscoelastic properties of the tissue with high resolution due to its high accuracy in displacement measurement at the micrometer level. High frame rate ultrasound strain imaging, which is the essential part of the LAM method, allows capturing the local viscoelastic parameters *in vivo* like breast tissue. The main components of the LAM test are a compression mechanism that is used to exert an approximately step-force on the tissue and a high-frame rate ultrasound system for monitoring the internal local strain responses. In other words, a step-force is used as a stimulus for a certain amount of time, and the transient strain response, which is governed by viscoelastic properties of the medium, is monitored by analyzing a sequence of radiofrequency (RF) data during the excitation^31,32^. In the LAM method the local tissue behavior in the sub-Hertz frequency range is used because at this frequency range the local biphasic behavior of tissue is more evident compared to other frequency ranges^33–37^.

## Results

### Viscoelastic gel phantom

Gel phantom can be used as a simplified mechanical model for breast tissue. Gel consists of collagen type I matrix that is saturated in water^38^, similar to breast tissue. The matrix peptide chain in this type of collagen is responsible for the dense electric charge associated with the hydrophilic properties of collagen fibers and culminates in the viscoelastic properties of the medium^39^. The same mechanism can happen in breast tissue in which the glycoproteins are responsible for viscoelastic properties due to their hydrophilic nature^39^. The viscosity of the fluid for these two media, however, is different. The solid matrix in both gel and tissue makes a porous structure which helps move the fluid when the medium is compressed or under load^40^. Fluid viscosity is responsible for the viscoelastic response of the media or tissue. In addition the hydrogen crosslinks between the fibers in a solid matrix can trigger the viscoelastic response^40^.

To verify the performance of the proposed model-free method, a viscoelastic inclusion phantom was made. The inclusion part of the phantom was made with 25.14 grams of gelatin (Sigma-Aldrich, St. Louis, MO), 60 ml propylene glycol (Sigma-Aldrich, St. Louis, MO), and 4 grams cellulose (ultrasound scattering; Sigma-Aldrich) in distilled water for a total volume of 300 ml. The background part of the phantom was made with 32.3 grams gelatin, 30 ml Vanicream Lite (Pharmaceutical Specialties, Inc., Rochester, MN), 6 grams cellulose (ultrasound scatterer; Sigma-Aldrich) and potassium sorbate (preservative; Sigma-Aldrich) in distilled water with a total volume of 600 ml^30^. The inclusion phantom dimensions were 7.5 cm × 5.5 cm × 5.5 cm (L × W × H), with the cylindrical inclusion having a diameter of 1.5 cm.

**Figure 1:**
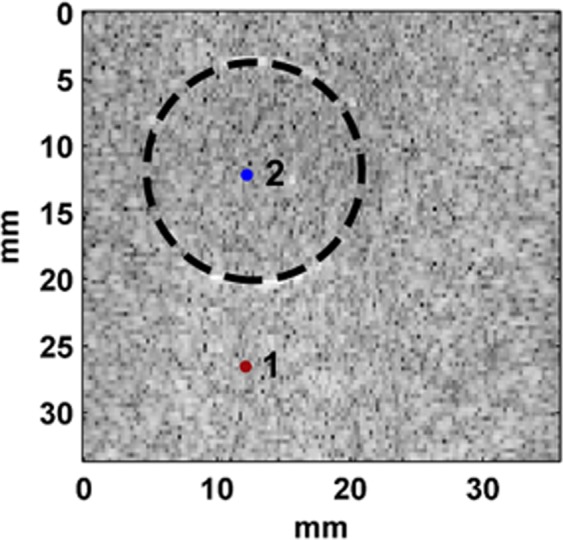
Ultrasound Verasonics B-mode image of the gel phantom with the inclusion part indicated with a dashed line. 1 = a point in the background; 2 = a point in the inclusion.

**Figure 2:**
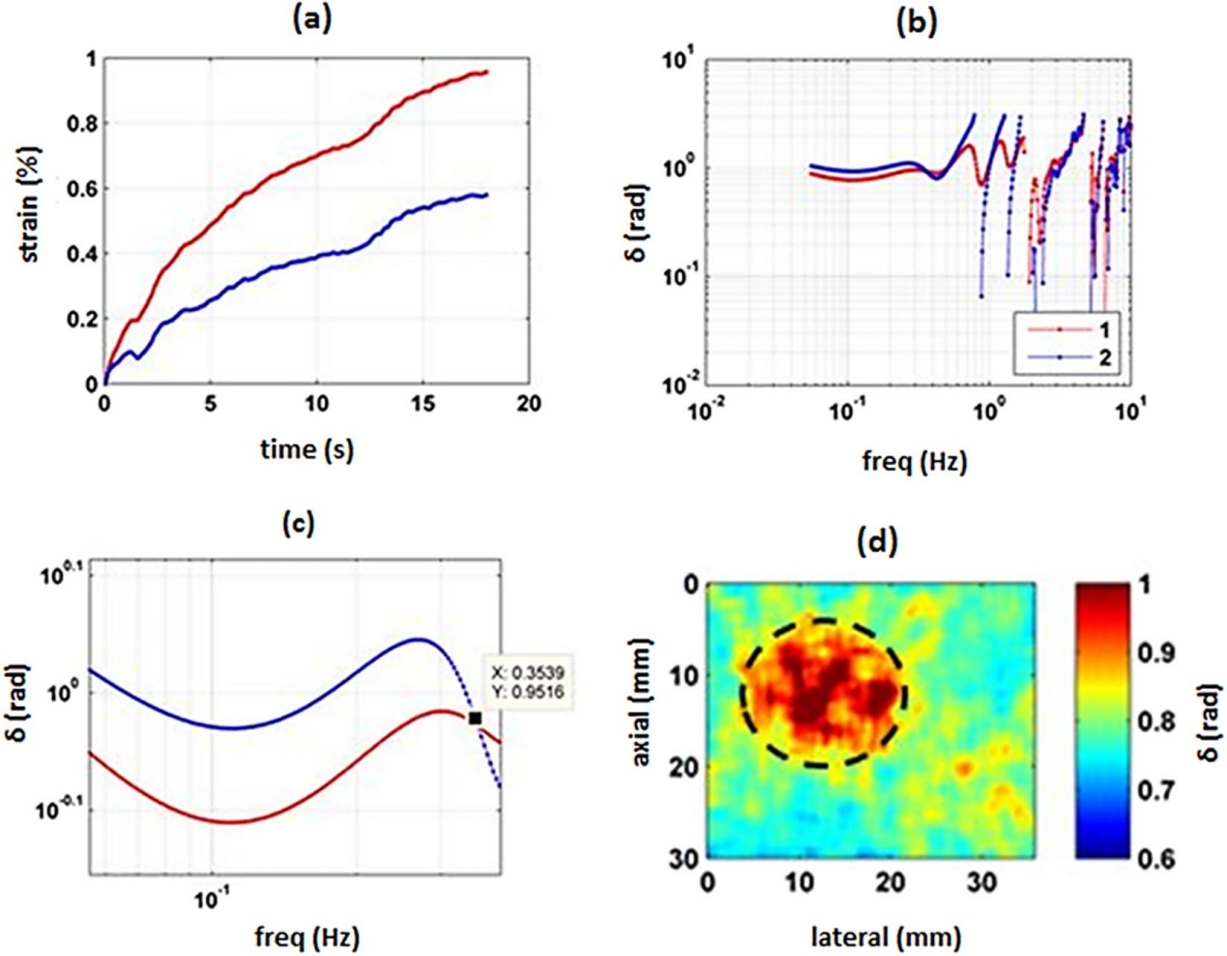
Profiles obtained on the inclusion gel phantom. (**a**) Temporal strain profile of point 1 (background, red) and point 2 (inclusion, blue) in the phantom imaged in Fig. 1. (**b**) Loss angle profile of point 1 (background, red) and point 2 (inclusion, blue) in a frequency range less than 10 Hz. (**c**) Loss angle profile of point 1 (background, red) and point 2 (inclusion, blue) in a frequency range less than 0.35 Hz. (**d**) Viscoelastic map produced at 0.033 Hz based on the LAM method.

An ultrasound B-mode image of the gel phantom can be seen in Fig. 1. For demonstration purposes, two points were selected: point 1 in the background and point 2 in the inclusion. The results of the temporal strain profile for these points can be seen in Fig. 2a. Loss angle profiles for these 2 points were obtained in the frequency range less than 10 Hz (Fig. 2b) and less than 0.35 Hz (c). Similar to the above mentioned 2 points the viscoelastic map created by processing all the spatial points at a frequency of 0.033 Hz using the LAM method as shown in Fig. 2(d).

### ***In vivo*** patient study

#### **Patient study**

The phantom study results encouraged us to apply the LAM method on breast patients. The patient study was approved by the Institutional Review Board of the Mayo Clinic, Rochester MN, and informed consent was signed by each enrolled patient. This study was also compliant with the Health Insurance Portability and Accountability Act (HIPPA) in the Mayo Clinic.

A total of 156 female patients with visible breast lesions in US images were recruited at Mayo Clinic from November 2014 to September 2016. The data from the first five patients were used to test the device and algorithm and were eliminated from the final study. Thus, a total of 151 breast patients were included in the study. Amongst them after applying the motion compensated cross-correlation metric (MCCC), 45 patients were selected for analysis. The rest of the patients were rejected. The mean patient age was 56 ± 15 years within the age range of 25–85 years.

**Figure 3:**
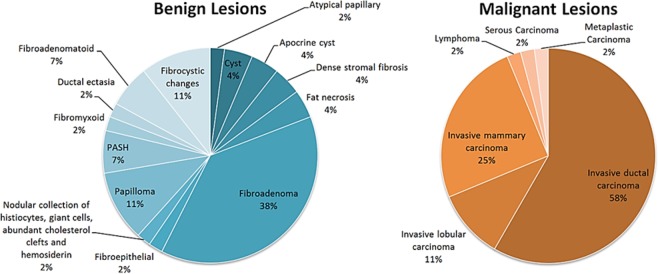
Distribution of lesion type in patient population.

Breast lesions were categorized using the Breast Imaging Reporting and Data System (BI-RADS). Figure 3 illustrates the distribution of lesion type in this patient population and Table 1 shows the BI-RADS distribution among them.

**Table 1:** Distribution of patients.

BI-RADS	2	3	4	5	6
Number of patients	0	8	102	42	4

**BI-RADS determination of lesions.** The BI-RADS value was determined for each lesion according to the sonographic features found in clinical US images obtained during a clinical procedure at Mayo Clinic. The patient’s eligibility for biopsy was decided based on this value. For BI-RADS 5 and 6, a biopsy is always prescribed; as such, cases are highly suggestive of malignancy. A BI-RADS value of 3 or 4 is challenging as this covers a wide range of suspicion, including low, intermediate, and moderate. In our study, all the BI-RADS 3 and BI-RADS 4 were biopsied.

The LAM technique was performed after determination of the lesion BI-RADS and location, and prior to the biopsy procedure.

**Histology.** Surgical excision biopsy or US-guided core needle biopsy was performed as a part of clinical care and the histology results for all patients in this study were available. In the latter cases, five core biopsy samples of each lesion were acquired by one of our certified radiologists. An experienced Mayo Clinic pathologist with more than 15 years of experience provided the histopathological diagnosis. Surgical histopathology was considered conclusive over core needle biopsy.

**LAM results.** As mentioned before, 156 patients were recruited for the study. The statistical results related to the whole patient population are depicted in supplementary part of this paper. Amongst those patients, 45 patients passed, the criterion set by MCCC metric. Only these female patients with a suspicious breast lesion (15.93 ± 8 mm) visible in ultrasound images were considered in this study. The mean and median age for this group was 56 and 55 years, respectively. The youngest participant was 25 and the oldest was 85 years old. All the patients underwent a biopsy procedure after the LAM test.

**Figure 4:**
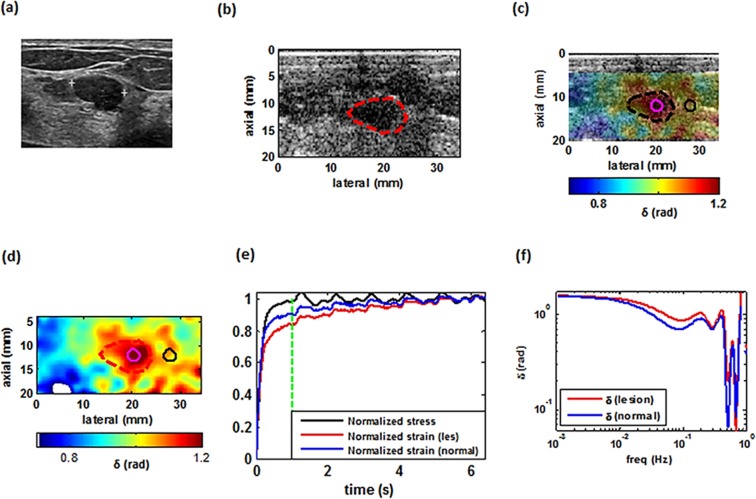
Imaging of a benign tumor. (**a**) Ultrasound clinical B-mode image of a benign tumor. (**b**) Ultrasound Verasonics B-mode image of the same tumor. (**c**) 2D color map of the loss angle overlay on the B-mode image produced at 0.033 Hz based on the LAM method. (**d**) 2D color map of the loss angle produced at 0.033 Hz. (**e**) Normalized strain temporal behavior of the two specified points in (**d**) accompanied with a normalized stress profile. **(f)** Spectral behavior of the two specified points in (**d**) in a frequency range less than 1 Hz.

**Figure 5:**
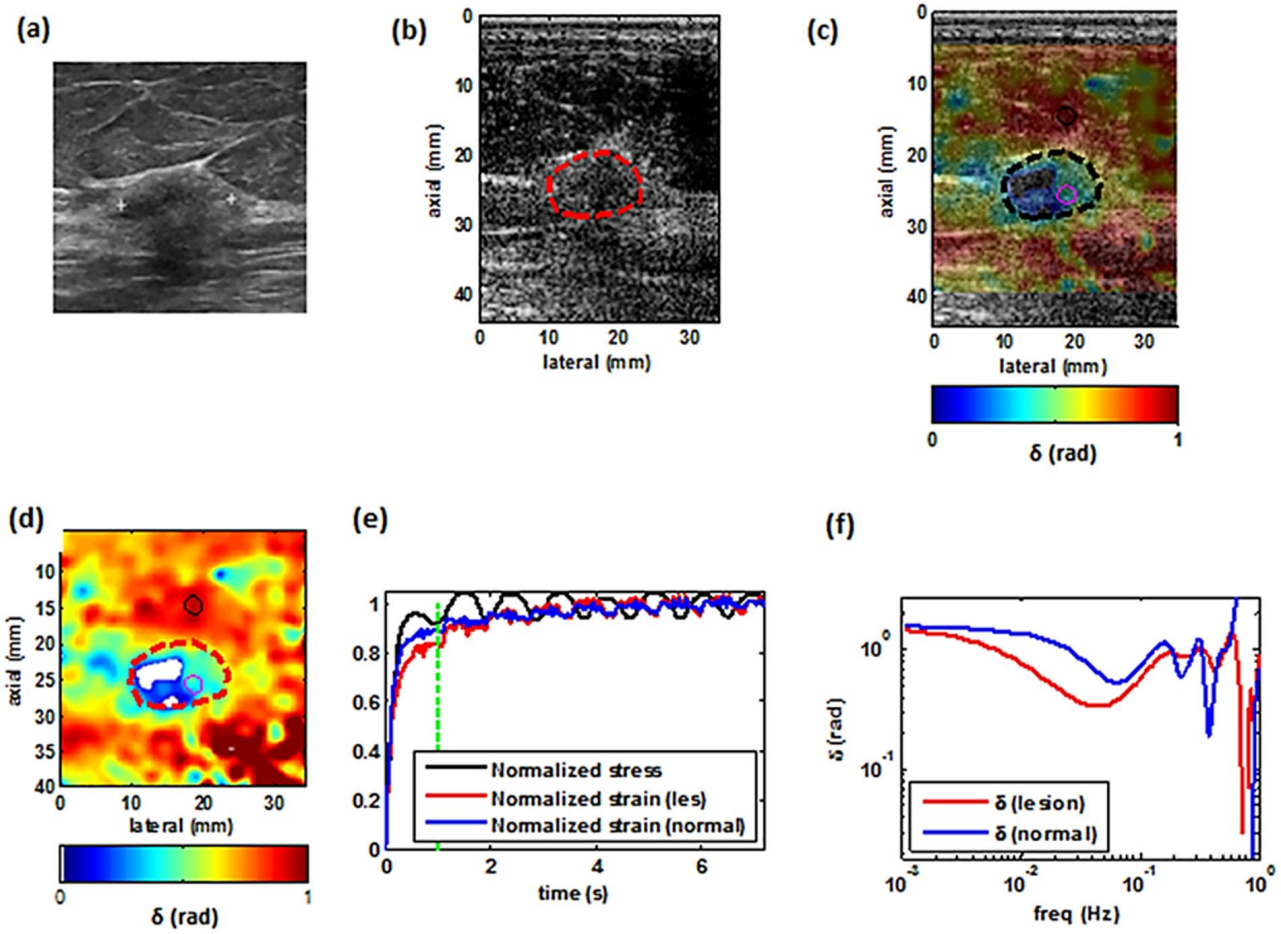
Imaging of a malignant tumor. (**a**) Ultrasound clinical B-mode image of a malignant tumor. (**b**) Ultrasound Verasonics B-mode image of the same tumor. (**c**) 2D color map of the loss angle overlay on the B-mode image produced at 0.18 rad/s (~0.03 Hz) based on the LAM method. (**d**) 2D color map of the loss angle produced at 0.03 Hz. (**e**) Normalized strain temporal behavior of two specified points in (**d**) accompanied with the normalized stress profile. **(f)** Spectral behavior of two specified points in (**d**) in a frequency range less than 1 Hz.

**Figure 6:**
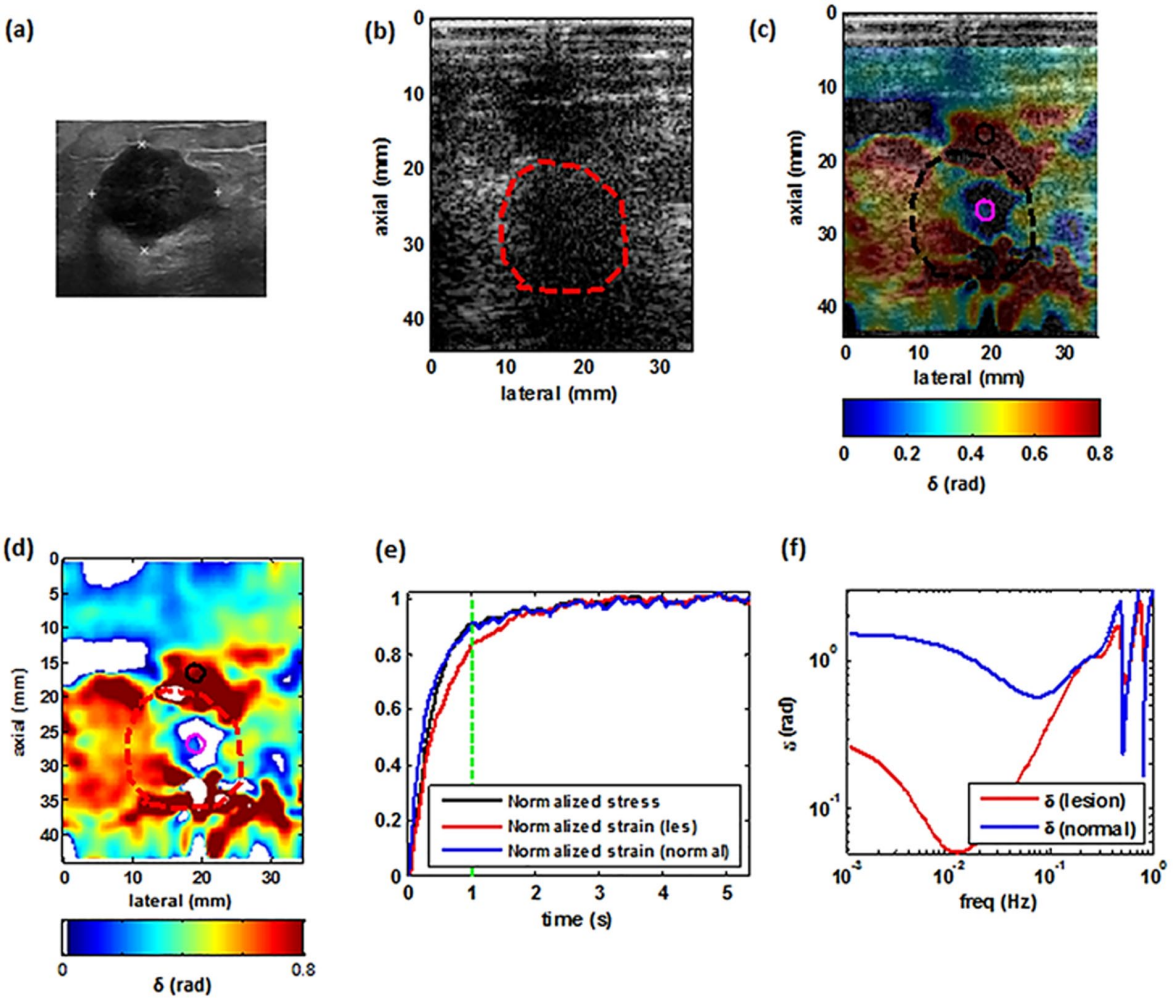
Imaging of a malignant tumor. (**a**) Ultrasound clinical B-mode image of a malignant tumor. (**b**) Ultrasound Verasonics B-mode image of same tumor. (**c**) 2D color map of the loss angle overlay on B-mode image produced at 0.033 Hz based on the LAM method. (**d**) 2D color map of the loss angle produced at 0.033 Hz. (**e**) Normalized strain temporal behavior of two specified points in (**d**) accompanied with the normalized stress profile. **(f)** Spectral behavior of two specified points in (**d**) in a frequency range less than 1 Hz.

**Figure 7:**
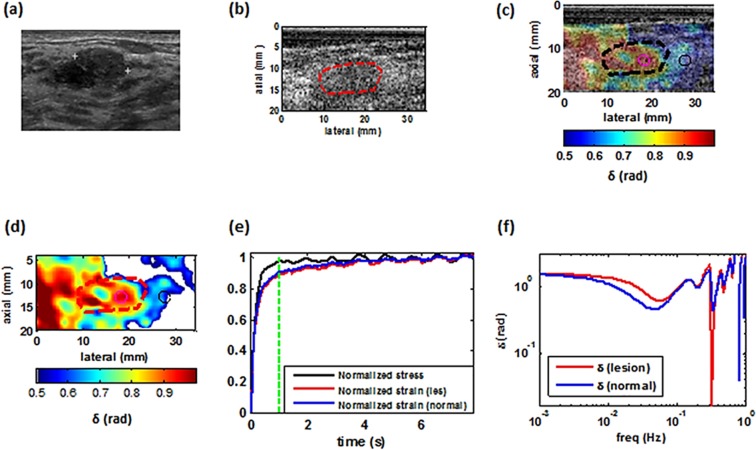
Imaging of benign tumor. (**a**) Ultrasound clinical B-mode image of  benign tumor. (**b**) Ultrasound Verasonics B-mode image of the same tumor. (**c**) 2D color map of the loss angle overlay on the B-mode image produced at 0.033 Hz based on the LAM method. (**d**) 2D color map of the loss angle produced a0.033 Hz. (**e**) Normalized strain temporal behavior of two specified points in (**d**) accompanied with the normalized stress profile. (**f**) Spectral behavior of two specified points in (**d**) in a frequency range less than 1 Hz.

Figures 4–7 illustrate examples of the loss angle maps of various malignant and benign lesion types. In all of these images, the LAM map is created at the frequency of 0.033 Hz. for each patient. we examined the clinical image, the corresponding B-mode image as seen using the I-Q data from the programmable ultrasound machine (Verasonics), an overlay of the estimated loss angle, a loss angle map with unreliable areas excluded (white areas in Figs 4(d),5(d),6(d) and 7(d)), a graph of the normalized applied stress, and representative temporal normalized strain curves from the lesion and normal tissue areas and corresponding estimated loss angle as a function of frequency.

**Figure 8:**
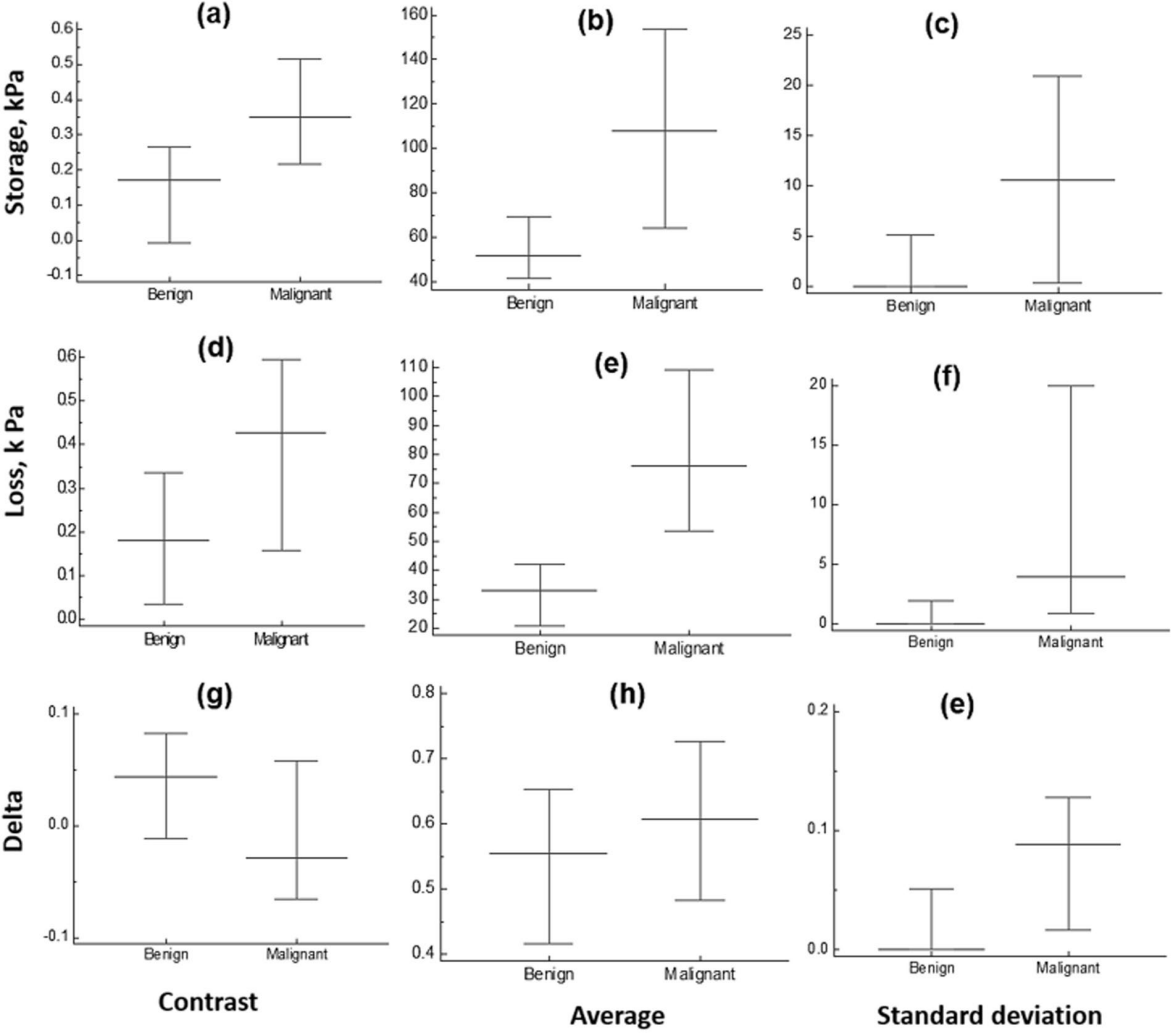
Summary of loss angle modulus parameters: Storage, Loss and Delta in 45 breast lesions.

Among 45 patients, 27 cases were diagnosed as benign and 18 as malignant. LAM method, Eqs (1 and 2), is used to estimate the complex modulus parameters (storage, loss) as was illustrated in Figs 4–7 for delta, δ. The overall results are depicted in following figures. Figure 8 shows the result based on the contrast, average and standard deviation measured in tumor part.

**Figure 9:**
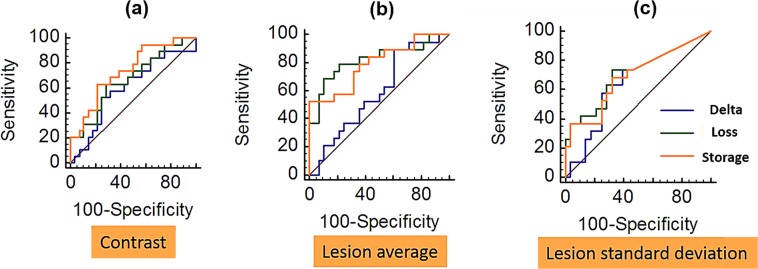
ROC curve for 45 breast lesions. (**a**) ROC based on measuring the contrast. (**b**) ROC based on lesion average (**c**) ROC based on standard deviation.

In the next step, the Logistic regression is applied on all of these aforementioned parameters shown in Fig. 8. The relevant ROC (Receiver Operating Characteristic) curve for each analysis is illustrated in Fig. 9.

The measured sensitivity is 77.8%, and the estimated specificity is 96.3%. The accuracy comes to 88.9%. The Area under Curve, AUC, is 0.94. The standard error is 0.04. 95% Confidence interval is 0.82 to 0.99. Detailed information is shown in Table 2 in the supplementary part. It should be noted that, the BI-RDAS results were considered in the aforementioned statistical results.

## Discussion

Due to biphasic properties of soft tissue, there is always some phase lag between applied stress and the resulting strain. In addition to storage and loss modulus magnitude, the LAM method can leverage the phase lag between these two parameters to study the local viscoelastic properties of living tissue in more details^31,41^. The resulting contrast based on the storage and loss modulus or their ratio can be even more conspicuous in a lower frequency range. Mizuno *et al*.^11^ reported that nonequiliburium fluctuations were observable in cytoskeletal networks in a frequency range less than 10 Hz using an active rheometer. Implementing micro rheology on cardiac thin filaments also demonstrated that, in a frequency range less than 10 Hz, both the storage and loss modulus had been elevated by increasing the Ca^2+^^12^. In addition, it has been shown that in living cells, more complex behavior at low frequency ranges can occur^42^. Rheometry techniques also have been used on variety of tissue samples to validate the newly developed elastography techniques^43–45^. In the most recent study, the rheometer results confirmed the higher contrast in viscosity in the frequency range less than 1 Hz^45^.

Benign breast tumors are generally more viscous with higher phase lag, Fig. 8(g), than the malignant ones, particularly with fibroadenoma cases^31,32^. The extra cellular matrix (ECM) is mainly made of a collagen fiber network to which proteoglycan molecules are attached^46–49^. The primary part of these molecules is the dense and hydrophilic sulfate groups^50^. Increasing the concentration of proteoglycan, and consequently the surrounding water molecules in ECM, can cause an increase in viscosity^31^. The collagen fibers in ECM are interconnected toward their ends but sparsely connected in the middle, with strong covalent cross-links, which are responsible for the elastic response of the tissue to stress^31^.

According to electron microscopy observation of malignant breast lesions such as infiltrating ductal carcinoma, the number of sulfated proteoglycan molecules, which are mainly responsible for viscosity, declines at least five times per unit in connective tissues. Thus, while viscosity decreases in malignant lesions, they can be stiffer than surrounding tissue due to increased collagen fibers and covalent cross-links^46,49,50^. There is, however, no sign of elevation in viscosity in the formation course of this kind of lesions^46,49^. The common feature in benign solid lesions, such a fibroadenomas, is higher collagen density compared to surrounding tissues. An increase in the number of fibers leads to a greater number of proteoglycan molecules and decreased inter-fiber distance. The lower distance between collagen fibers will cause a greater H-bonded cross-link density. Generally, H-bonds help in stabilizing the ECM matrix by keeping the helical shapes within fibers. This functionality of H-bonded cross-links has an important role in creating an elastic restoring force immediately after being stressed. However, these fragile bonds break and reform during the stress application and some of the strain energy is dissipated. This causes the strain response delay in returning to the initial steady state, (usually in less than 5 seconds^31^). In other words, this is the reason for viscoelastic response of the external compression, which occurs at a very low frequency range based on its retardation time. Thus, collagen density determines the strength and density of inter-fiber H-bonded links. Some fibrous tumors are not necessarily stiffer than surrounding regions, but due to higher cross-link density, can create a longer delay for a full strain response^32,46–50^. This phenomenon is the basis of viscoelastic methods like LAM method. Thus, in the case of fibroadenomas and other collagenous benign lesions, the lesion can be stiffer than the surrounding tissues but they are more viscous in contrast to malignant lesions^31,32,49^ Fig. 8(g) also showed that phenomena. To observe such differences, it is necessary to evaluate tissue’s viscoelastic response in a period of 1–10 s, which corresponds to a frequency range less than 1 Hz^32,34^.

This work on breast patients showed that considering the viscoelastic behavior of tissue by estimating the complex elasticity parameters like storage, loss and delta in an ultra-low frequency range could provide reliable biomarkers for differentiation of benign and malignant tumors in breast patients. The distinctive characteristic of the LAM method is its sensitivity to viscoelastic properties of breast tissue in this range of frequency. This characteristic correlates well with the biochemical construction of benign and malignant breast lesions. The sensitivity and specificity of this method in differentiation of benign and malignant lesions were 77.8% and 96.3% respectively.

A possible contributing factor in misclassifications is measurement error. Lesion mobility can adversely affect the quality of recorded data. Patient motion during a test can also negatively affect data quality. Some patients were not able to hold their breath for the entire duration of data collection, which resulted in tissue motion. Cardiac motion was not considered as a major source of error as the frequency of such motion is around 1 Hz, which is somewhat outside the frequency band of these ultra-low frequency range calculation, less than 0.05 Hz.

Due to all aforementioned reasons, we tried to apply more restrictions on our motion measurement algorithm. The initial patient population that was considered for this study was 156 patients. The statistical outcomes of these patients are shown in Figs S1 and S2, Tables S1 and S2 in supplementary part. However, applying MCCC algorithm in order to detect the out-of-plane and non-axial motion while doing compression was a great assistance to identify cases with mostly axial compression with minimal out-of-plane motions. While this is done retrospectively, if implemented to operate in real-time, such metric can be utilized to ensure proper lesion compression and data collection. Reconsidering these data and using MCCC algorithm resulted in 45 cases out of 156 cases as they have been discussed already. In addition, a multi-parameters analysis using the features obtained from the all creep data (i.e. storage, loss modulus and delta) in conjunction with contrast, average and standard deviation measurement, provided and enhanced classification of the breast tumors in this study, Figs 8 and 9. Future studies would include expanding the cohort and studying the sources of error in our measurement. This multi factorial analysis is another advantage of the LAM method.

LAM can be utilized in conjunction of other ultrasound-derived lesion characteristics such as BI-RADS and size. Comparing Tables 2 and 3, with and without considering BI-RADS respectively shows that both the specificity and sensitivity have been elevated from 89.29% to 96.3% and 68.42% to 77.78% respectively. The combined value of LAM and ultrasound imaging features promise better diagnosis without requiring more expensive and contrast-based imaging modalities such as contrast-enhanced magnetic resonance imaging.

**Table 2:** Classification table when using MCCC, BI-RADS number and tumor size (Cut-off value = 0.5).

Actual group	Predicted group		Percent correct
	0	1	
Benign cases	26	1	96.30%
Malignant cases	4	14	77.78%
Percent of cases correctly classified			88.89%
**ROC curve analysis**			
Area under the ROC curve (AUC)		0.938	
Standard Error		0.039	
95% Confidence interval		0.824 to 0.988

**Table 3:** Classification table when using MCCC but not BI-RADS number or tumor size (Cut-off value = 0.5).

Actual group	Predicted group		Percent correct
	0	1	
Benign cases	25	3	89.29%
Malignant cases	6	13	68.42%
Percent of cases correctly classified			80.85%
**ROC curve analysis**			
Area under the ROC curve (AUC)		0.868	
Standard Error		0.056	
95% Confidence interval		0.738 to 0.949	

Another important aspect of this study is the automated ROI (Region of Interest) selection to remove the subjectivity associated with manual selection of data processing. In other words, all of the patient data was processed in a consistent manner without human input for selecting the region of interest or changing other parameters involved in data processing. First, the eligibility of data was tested for out-of-plane motions and then the processing began automatically either in selecting the lesion from normal part or in setting the creep legitimate time duration in which the monotonous increasing of compliance curve was counted. This procedure went through all the collected patient data.

It should be noted that as the results show, the LAM method could be a very effective method in the context of non-invasive diagnostic/prognostic mechanical testing especially for soft tissue; however, it may not be a solid candidate for mechanical property estimation in general.

In addition, the LAM method was observed to present a solid contrast between benign and malignant lesions at frequency range less than 0.033 Hz, however it needs more comprehensive studies to find the optimum frequency with highest contrast in this ultra-low frequency range. This would be explored in future work.

## Methods

### **LAM technique**

In this patient study, an automated compression device was used to apply a ramp-and-hold force excitation for a duration of time automatically set based on monotonically elevation of creep response. The ultrasound probe is part of this device for recording the viscoelastic response of the underlying tissue. This device is portable, light-weight and easy to use for patient studies to explore the tissue dynamics under external stress^30^.

For the gel study, 8 N force with the speed of 16 N/s ramp was used. For the patient study, 2–4 N force with 8–12 N/s ramp were applied. To monitor the local viscoelastic response, an ultrasound system (Verasonics, Inc., Kirkland, WA, USA) with a linear array transducer (L11-4v, Verasonics, Inc., Kirkland, WA, USA) was used. In these experiments, the ultrasound center frequency was 6.43 MHz and the frame rate was 200 Hz during the acquisition time of 12 seconds.

To track the fast tissue deformation under compression, a two-dimensional autocorrelation method was employed for a particle velocity calculation from adjacent frames. The integration of the particle velocity in time resulted in a displacement estimation^51^. After performing the displacement estimation for all consecutive IQ data, the gradient of the resulting displacement was computed to measure the corresponding local strain^30^.

For patient studies, an experienced sonographer with more than 28 years of experience in breast ultrasound assisted with manual delineation of the lesion in B-mode US images obtained from the Verasonics system at the beginning of each patient data acquisition. Using the radiofrequency data, the loss angle maps were reconstructed offline at a frequency range less than one Hertz. Supplementary video is available online.

#### **Dynamic complex measurement**

It was shown that the dynamic complex modulus in the frequency domain, E*(*ω*), can be directly derived using the strain time data (local creep response) in a model-free fashion with some assumption about the experimental creep response^13,52,53^, Eq. 1.

1 $${E}^{\ast }(\omega )=\frac{i\omega }{i\omega J(0)+\frac{{e}^{-i\omega t(N)}}{\eta }+{\sum }_{n=1}^{N}(\frac{J(n)-J(n-1)}{t(n)-t(n-1)})({e}^{-i\omega t(n-1)}-{e}^{-i\omega t(n)})},n=1:N$$

where J(n) represents the strain data sampled at time point with index *n*. The parameter $\eta$ represents the steady state viscosity which is estimated by extrapolation of strain data to *t* → *∞*.

Hence, the loss angle can be directly derived as

2 $$\delta =arctan(\frac{{E}_{l}^{\ast }(\omega )}{{E}_{s}^{\ast }(\omega )})$$

where ${E}_{l}^{\ast }(\omega )$ and ${E}_{s}^{\ast }(\omega )$ are the imaginary and the real parts of the complex shear modulus, corresponding to the loss and storage modulus, respectively, and δ is the loss angle which represents the phase difference between the storage and loss modulus of the medium due to its viscoelastic properties^13,52^.

#### **Measurement**

**Strain Quality Assessment, SQA.** Our main assumption in this study is the linear viscoelastic behavior of the material being tested (phantoms or tissue). Applying an approximate step force results in a temporal strain response, or creep response, which increases monotonically. This behavior is called a normal strain response. Applying this method for *in vivo* studies, however, requires introduction of additional constraints to avoid estimation of unrealistic values. Here, we define a new parameter as Strain Quality Assessment (SQA). SQA verifies two parameters: the total strain and the slope of the final part of the creep response. For each point in the viscoelasticity map reconstruction domain, SQA assigns a value of one for points that have both a positive total strain value and positive final slope, and zero if either of these criteria is violated. All the white areas in Figs 4(d), 5(d), 6(d) and 7(d) are excluded due to SQA.

**Contrast:** Contrast refers to a comparison between features of a lesion to those of the surrounding, or background, tissues. For *in vivo* studies, measuring contrast parameters is important for diagnostic purposes. In some cases, measuring the contrast is even more important than measuring the parameter values^32^. In order to measure contrast we use the Eq. (3).

3 $$Contrast=\frac{{S}_{lesion}-{S}_{background}}{({S}_{lesion}+{S}_{background})/2}=\frac{Difference}{Average}$$

In this equation *S*_*lesion*_ is the mean value in the lesion area and *S*_*background*_ represents the mean value of the normal background tissue surrounding the lesion. To measure contrast, the mean value of S for the lesion and background parts should be determined. This is performed by first determining the lesion boundaries from a registered B-mode image within the region of interest (ROI). The automatic ROI was applied to remove the subjectivity about lesion and normal tissue values. In addition, the creep duration was selected automatically based on monotonic increasing compliance curve. As it has been emphasized before all data in this manuscript was analyzed at 0.033 Hz.

**Rejection of data based on MCCC metric:** The displacement field obtained from the phase sensitive speckle tracking was utilized to stretch each frame back to its original location, as explained in^54^. Briefly, the normalized cross correlation was performed between the pre-compressed and motion-compensated post-compressed echoes in the lesion area. This value served as a quality metric to assess the uniaxiality of the induced motions. Given substantial decorrelation that may occur during compression, a minimum normalized cross correlation value of 10% was chosen to declare a successful uniaxial deformation. Using this criterion, cases/acquisitions that did not meet a required threshold value of MCCC metric were excluded from the analysis.

#### **Statistical Analysis**

MedCalc Statistical Software version 15.8 (MedCalc Software bvba, Ostend, Belgium; https://www.medcalc.org; 2015) was employed for statistical analysis. The receiver operator curve (ROC) was used to find the best diagnostic discrimination threshold for the estimated loss angle contrast in comparison with the pathology outcomes. The confidence intervals for sensitivity and specificity were found using bias-corrected and accelerated bootstrapping of 1000 trials. Resulting sensitivity, specificity and area under the curve were reported.

The Wilcoxon analysis was performed with 95% confidence interval to represent a statistically significant difference for all analysis. All multi-parameter analyses were performed using a logistic regression method in MedCalc.

## Supplementary information

Supplementary
