# Supplementary material for: Viscoelastic biomarker for differentiation of benign and malignant breast lesion in ultra- low frequency range
Source: Sci Rep. 2019 Apr 5;9:5737. doi: 10.1038/s41598-019-41885-9 (PMC6450913; doi:10.1038/s41598-019-41885-9)
Supplement: Supplementary file 1 — Supplementary [file 41598_2019_41885_MOESM1_ESM.pdf]

Viscoelastic biomarker for differentiation of benign and malignant breast lesion in ultra-low frequency range

*Alireza Nabavizadeh[1,2,4], Mahdi Bayat[1,4],Viksit Kumar[1], Adriana Gregory[1], Jeremy Webb[1], Azra Alizad[1,3], Mostafa Fatemi[1]

Department of Physiology and Biomedical Engineering, Mayo Clinic College of Medicine, Rochester, Minnesota, USA[1].
Biomedical Informatics and Computational Biology, University of Minnesota[2]
Department of Radiology, Mayo Clinic College of Medicine, Rochester, Minnesota, USA[3].

*Correspondence to A. N. (email: an2801@columbia.edu)
Equal contribution[4]

Supplementary Figures

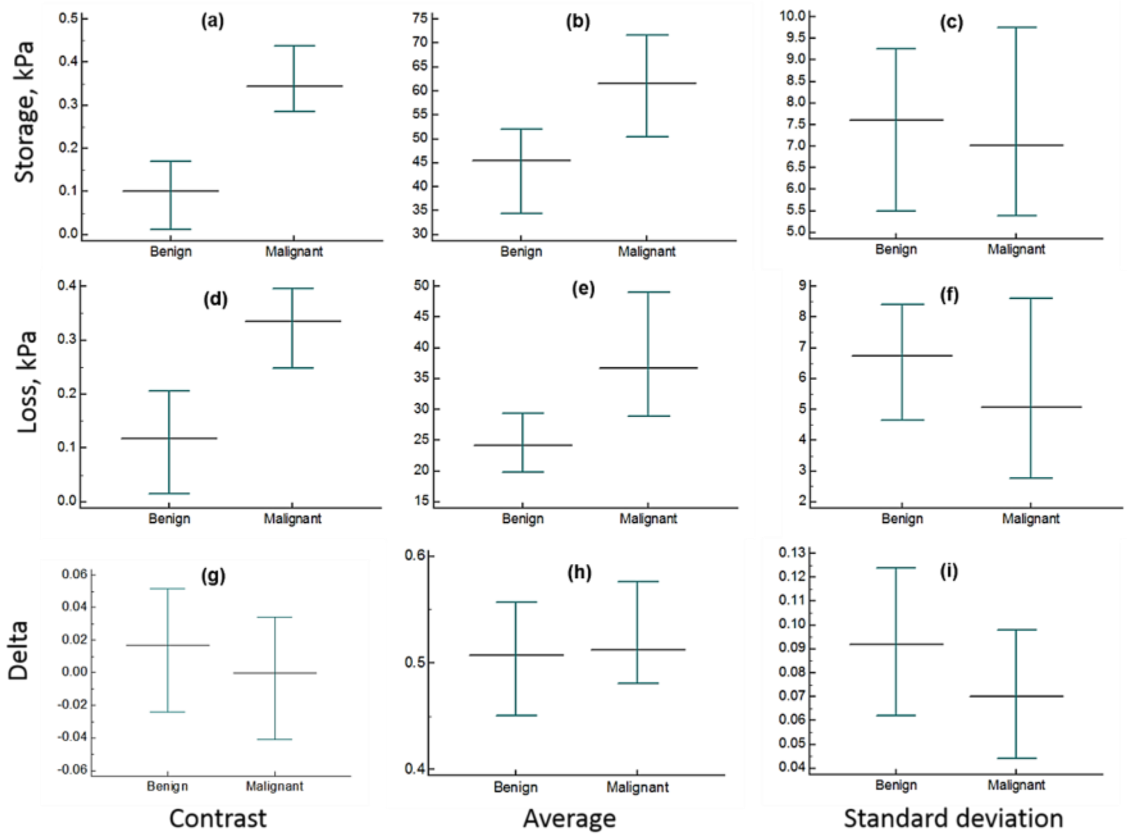

Figure S1. Summary of loss angle modulus parameters: Storage, Loss and Delta in 156 breast lesions without considering MCCC method.

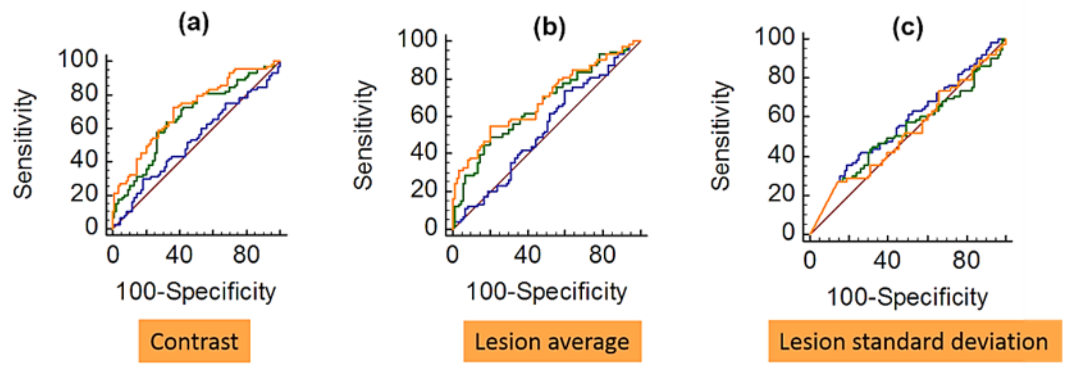

Figure S2. ROC curve for 156 breast lesions without considering MCCC method. (a) ROC based on measuring the contrast. **(b)** ROC based on lesion average. **(c)** ROC based on standard deviation.

Supplementary tables

Table S1. Distribution of patients with no MCCC, BI-RADS and tumor size
(Cut-off value p=0.5)

Actual group	Predicted group		Percent correct
	0	1	
Benign cases	67	16	80.72 %
Malignant cases	32	41	56.16 %
Percent of cases correctly classified			69.23 %

ROC curve analysis

Area under the ROC curve (AUC)	0.779
Standard Error	0.037

Table S2. Distribution of patients with no MCCC, with BI-RADS and tumor size
(Cut-off value p=0.5)

Actual group	Predicted group		Percent correct
	0	1	
Benign cases	72	8	90.00 %
Malignant cases	21	47	69.12 %
Percent of cases correctly classified			80.41 %

ROC curve analysis

Area under the ROC curve (AUC)	0.882
Standard Error	0.027
95 % Confidence interval	0.818 to 0.929
